# Surface Coatings Modulate the Differences in the Adhesion Forces of Eukaryotic and Prokaryotic Cells as Detected by Single Cell Force Microscopy

**DOI:** 10.1155/2019/7024259

**Published:** 2019-04-01

**Authors:** Philipp Wysotzki, Jan Gimsa

**Affiliations:** Department of Biophysics, Faculty of Natural Sciences, University of Rostock, 18057 Rostock, Germany

## Abstract

Single cell force microscopy was used to investigate the maximum detachment force (MDF) of primary neuronal mouse cells (PNCs), osteoblastic cells (MC3T3), and prokaryotic cells (*Staphylococcus capitis* subsp.* capitis*) from different surfaces after contact times of 1 to 5 seconds. Positively charged silicon nitride surfaces were coated with positively charged polyethyleneimine (PEI) or poly-D-lysine. Laminin was used as the second coating. PEI induced MDFs of the order of 5 to 20 nN, slightly higher than silicon nitride did. Lower MDFs (1 to 5 nN) were detected on PEI/laminin with the lowest on PDL/laminin. To abstract from the individual cell properties, such as size, and to obtain cell type-specific MDFs, the MDFs of each cell on the different coatings were normalized to the silicon nitride reference for the longest contact time. The differences in MDF between prokaryotic and eukaryotic cells were generally of similar dimensions, except on PDL/laminin, which discriminated against the prokaryotic cells. We explain the lower MDFs on laminin by the spatial prevention of the electrostatic cell adhesion to the underlying polymers. However, PEI can form long flexible loops protruding from the surface-bound layer that may span the laminin layer and easily bind to cellular surfaces and the small prokaryotic cells. This was reflected in increased MDFs after two-second contact times on silicon nitride, whereas the two-second values were already observed after one second on PEI or PEI/laminin. We assume that the electrostatic charge interaction with the PEI loops is more important for the initial adhesion of the smaller prokaryotic cells than for eukaryotic cells.

## 1. Introduction

The first quantitative studies on cell adhesion were performed with a simple wash assay. The samples were rinsed after different cell attachment periods. The remaining cells were stained and counted [1]. For the continuous registration of cell adhesion quartz crystal microbalances and thickness shear mode sensors have been developed. They permit statements to be made on the kinetics of cell adhesion using mass and complex elasticity effects as well as viscoelastic effects at the cellular interfaces to the sensor surface [2]. In interdigitated electrode structures (IDES), cell adhesion is registered via the electric impedance, which changes when the number of cells or their degree of spreading alters the electrochemically effective surface of the IDES [3–5].

Cell-to-surface adhesion forces can be studied using a variety of techniques, such as flow chambers or spinning discs [6, 7]. The spinning disc assay was developed for a better control of the applied forces. It permitted the application of defined shear forces to the cells on a surface by varying the rotational speed of the disc [8]. Nevertheless, in these techniques the mechanical detachment of cells depends not only on the strength, number, and distribution of the adhesion-mediating surface proteins, but also on the contact surface and geometry of the individual cells. Accordingly, the techniques lack precision in measuring the detachment force of individual cells from the surface examined [9].

However, some techniques, such as optical or magnetic tweezers, do overcome these limitations but are limited to forces below the nN range. Single cell force microscopy (SCFM) permits single-cell forces measurements in the 10 pN to 1000 nN range with the pico-Newton accuracy of atomic force microscopy (AFM) [7, 10]. However, the disadvantage of single cell measurements is their increased scatter, as the properties of the individual cells can differ greatly from the average properties of the cell population in the same culture dish [11, 12]. A large number of individual cells must be investigated in order to achieve a statistical significance [13]. This problem can be alleviated by examining different surfaces with the same single cell, reducing the number of experiments and making the results easier to compare. This approach was used, for example, by Canale et al. [9] and Yu et al. [13] who produced different surface coatings using techniques such as contact printing with successively applied stamps or molecular printers before examining the surfaces with the same single cell.

We used SCFM to compare the adhesion properties of osteoblast-like cell line (MC3T3), primary neuronal mouse cells (PNC), and* Staphylococcus capitis subsp. capitis *(Scc) cells on artificial surfaces. The adhesion behavior of neuronal cells or bone cells is important for the integration of implants into the surrounding tissue [14, 15]. The artificial surfaces need to promote the adhesion of somatic cells and prevent the adhesion of bacteria in order to prevent the formation of biofilms [16]. Clearly, the competing adhesion and proliferation between eukaryotic and prokaryotic cells on the same surface involve a complex hierarchy of consecutive processes. SCFM may capture the very early stages of these processes, which in our opinion are of enormous importance in order for somatic cells to win the “race for the surface” [16] and to be successful in colonizing the implant surface. In clinical applications, differentiations in the adhesion of somatic cells and microorganisms have been achieved by coating the surfaces with silver, vitamins, hyaluronic acid, antibiotics, or hydroxyapatite [17].

Surface coatings are a standard in cell culturing. In our SCFM experiments, we used glass-cover slips, which were sputter-coated with silicon nitride spots. One silicon nitride spot was used as a reference surface, while other spots were modified by PEI (polyethyleneimine), PEI/laminin, and PDL (poly-D-lysin)/laminin coating using conventional cell culture protocols [18, 19].

Ceramic silicon nitride is often used as an insulating layer in semiconductors and on conductive on-chip tracks of multielectrode arrays [3]. The positively charged, inorganic silicon nitride surfaces have been successfully used for cell culturing in lab-on-chip systems [20]. The PEI and PDL polymers are also positively charged, thus electrostatically favoring contact with the negatively charged cell surfaces. A number of authors have reported that PEI coatings improve cell adhesion for cell types such as PC12, HEK-283, and human osteoblast-like cells [21, 22]. PEI coatings are often used in neuronal cell cultures [23, 24]. However, it has been reported that PEI may be toxic in long-term cultures [25], a fact that we were unable to confirm in our long-term cell culture experiments (results not yet published). PDL is also a common coating in neuronal cell culture [18], as is laminin, since neuronal cells carry specific laminin-binding molecules [26, 27]. The laminin protein plays a role in cell adhesion and proliferation. It binds to ECM components such as collagens or nidogens [28]. Laminin is found in the basal laminae that form networks of web-like structures, which resist tensile forces.

In order to improve the comparability of the SCFM results, we looked for methods to attach prokaryotic and eukaryotic cells to the cantilever. Poly-dopamine is known to form strong and universally adhesive layers [29, 30]. It has been used before as an adhesion promoter in SCFM with prokaryotic cells [31, 32]. In previous SCFM experiments, we were also able to attach MC3T3 cells with poly-dopamine to SCFM cantilevers [33]. Here we found that poly-dopamine could also be used to attach PNCs. Using the same method for attaching the cell of all types to the cantilever, the number of influencing factors on the SCFM measurements was reduced. This improved the comparability of the determined maximum detachment forces (MDFs) for the three different cell types on different coatings. We were able to reduce the scatter in the overall MDF data by using silicon nitride as the reference surface. Our results concur with the idea that the initial adhesion of prokaryotic and eukaryotic cells on different surfaces is controlled by electrostatic interactions on time scales of less than one minute [34–36]. In our experiments, laminin acted as a geometric spacer that reduced direct cell contact with the charged surfaces. This is also true for PNCs, since the specific protein-mediated binding mechanisms are too slow to become effective.

## 2. Materials and Methods

### 2.1. Cell Culture and Preparation of Primary Cells

#### 2.1.1. Osteoblast-Like MC3T3 Cells (MC3T3)

The osteoblast-like MC3T3-E1 cells (in the following referred to as MC3T3) were obtained from the German collection of microorganisms and cell culture (DSMZ, Braunschweig, Germany). They were cultured in 25 cm^2^ cell-culture flasks (Greiner bio-one, Frickenhausen, Germany) in alpha MEM (ord. No. F 0925) supplemented with 1% penicillin/streptomycin (stock solution: 100 U/ml penicillin/100 *μ*g/ml streptomycin), and 10% fetal bovine serum (all purchased from Biochrom AG, Berlin, Germany). The incubator ensured 95% humidity in a 5% CO_2_ atmosphere at 37°C. Cells grown to confluence after approx. seven days were trypsinated (0.05% Trypsin with EDTA 0.02%, PAN Biotech GmbH, Aidenbach, Germany) and subcultured.

#### 2.1.2. Primary Neuronal Mouse Cells (PNCs)

NMRI mice at 16.5 days of pregnancy were sacrificed by cervical dislocation. The frontal cortex of the embryos was dissected and prepared for cell culture, as described in [37, 38]. The cells collected were transferred to cell culture dishes and cultured in D10 media (DMEM-high glucose, 10% horse serum, 1% Na-pyruvate) in an incubator with 10% CO_2_ and 95% humidity. A subcultivation step was performed to reduce contamination by unwanted cell types during the preparation procedure.

#### 2.1.3. Prokaryotic Scc Cells (Staphylococcus capitis subsp. capitis)

An undemanding prokaryotic organism was chosen for the experiments.* Scc *(ATCC 27840) is a biosafety level 1 organism and can be cultivated in standard media, but has still clinical relevance [39]. It is a gram-positive coccus with a diameter of 0.5 - 1.5 *μ*m, part of the skin flora, and able to form biofilms. After thawing, the bacteria were cultivated in planktonic form in casein soy peptone broth with 3% of yeast extract (Carl Roth, Karlsruhe, Germany) at 37°C for 24 h. Subsequently, casein yeast peptone agar plats (Fisher Scientific, Schwerte, Germany) were inoculated using an inoculation loop and incubated at 37°C for another 24 h.

### 2.2. Surface Preparation

**Figure 1:**
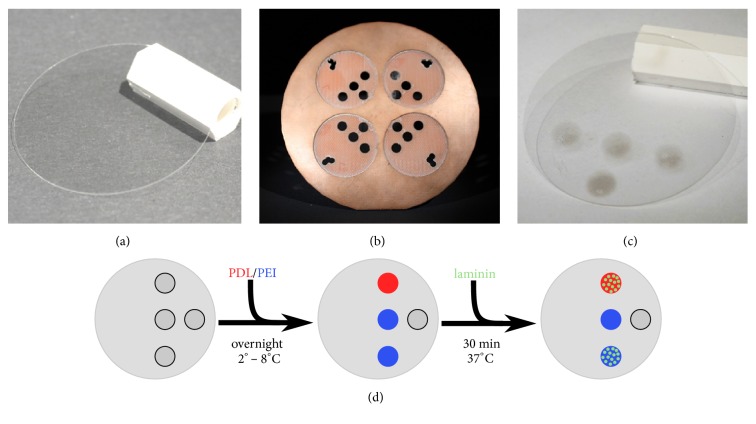
A 32-mm glass cover slip prior to silicon nitride coating (a); homemade sputter mask (b), and coated glass cover slip with four silicon nitride spots (c). (d) presents a schematic presentation of the coating procedure of the silicon nitride spots with PDL, PEI, and laminin. The fresh silicon nitride spots were incubated overnight with droplets of PEI (red) or PDL (blue) (left and center). Prior to the experiments, two spots were incubated with laminin (green) for 30 minutes (right).

Round 32-mm glass cover slips were purchased from Menzel Glas (Gerhard Menzel GmbH, Braunschweig, Germany). The cover slips were rinsed with acetone and cleaned in a plasma-cleaner prior to sputter coating (Zepto LF; Diener electronic GmbH, Ebhausen, Germany) at approx. 75 W power for 180 seconds. Four cover slips were placed in the homemade mask, which had four round indentations (Figures 1(a)–1(c)). Four 5-mm holes were drilled in one half of each indentation. An “L” like shape was drilled into the opposite side to facilitate identification of the coated side. Silicon nitride (Si_3_N_4_) was deposited by magnetron sputter deposition (PPS-90UV, Von Ardenne GmbH, Dresden, Germany) at 200 W for 150 seconds.

Prior to polymer coating, the glass cover slips were cleaned with Terg-a-zyme (Merck KGaA, Darmstadt, Germany), rinsed with water, and dried under a laminar airflow. The first layer was applied by adding 5-*μ*l droplets of 0.5% PEI in BORAT buffer, pH 8.5 (mean M_w_ 750,000, Merck KGaA, Darmstadt, Germany), or of 1 mg/ml PDL in ultra-pure water (mean M_w_ 70,000-150,000, Merck KGaA, Darmstadt, Germany) solution onto the silicon nitride spots and incubating overnight at 2 – 8°C. Prior to the SCFM measurement, the second layer was adsorbed by adding 5-*μ*l droplets of laminin solution (laminin-111, 0.5 mg/ml, Roche Diagnostics GmbH, Grenzach-Wyhlen, Germany) onto the spots (Figure 1(d)). After 30 minutes at 37°C, the coated cover slip was placed on the bottom of a 35 mm cell culture dish (TPP® AG, Trasadingen, Switzerland) and fixed with a small magnet. Coating material that had not been adsorbed was removed by rinsing with PBS.

### 2.3. Laminin Surface Coverage

Fluorescence microscopy was used to compare the laminin densities on PEI and PDL base coatings. In the experiments, the surface-bound laminin was bound by a primary rabbit antibody (Anti-Laminin, Merck KGaA, Darmstadt, Germany). The primary antibody was detected by a secondary antibody (Anti-Rabbit-IgG-Mega 485, Merck KGaA, Darmstadt, Germany) labelled with a fluorophore with an excitation wavelength of 485 nm and an emission wavelength of 552 nm. The fluorescence intensities of the labeled laminin molecules were examined under an Axio Observer A1 microscope (Zeiss, Oberkochen, Germany).

### 2.4. Cantilever Coating

Prior to each measurement, a fresh, tip-less cantilever (Arrow TL-1, Nanoworld, Neuenburg, Switzerland) was functionalized with poly-dopamine. To remove any possible contamination, the cantilever was cleaned by UV-ozone treatment (2x 8 W UV-C ozone fluorescent tube, Dinies Technologies GmbH, Villingendorf, Germany) for 300 s and later carefully submerged in a 100 *μ*l droplet of carbonate-buffer (pH 8). Then, 2 *μ*l of a freshly prepared dopamine hydrochloride solution (Sigma Aldrich, St. Louis, USA; 2 mg/ml DOPA-HCl, 5% acetic acid) was added. The formation of poly-dopamine was induced by increasing the pH to an alkaline condition, by adding 2 *μ*l of sodium hydroxide (2 M). The solution was incubated at room temperature for 25 minutes and then washed with a generous amount of PBS.

### 2.5. SCFM Measurements

The coated cover slip was placed into the Petri dish heater (PetriDishHeater™, JPK, Berlin, Germany) and transferred to the microscope stage of an Axio Observer A1 microscope (Zeiss, Oberkochen, Germany) that was part of the SCFM setup. The Petri dish was filled with 3 ml PBS and heated to 37°C. The cantilever was mounted on the AFM stage (CellHesion/Nanowizard II AFM, JPK Instruments, Berlin, Germany), placed on the microscope stage, and submerged in the Petri dish filled with PBS. For details see [40].

Before the SCFM measurements, the cantilever was calibrated above a noncoated area by the thermal noise method using the second harmonic frequency, which was approx. 7 kHz [41]. Then, 500 *μ*l of the PBS in the cell culture dish was exchanged for cell suspension. Shortly thereafter, the cantilever was aligned over a sedimented single cell and carefully approached. Mild pressure with a force of 2 to 6 nN was applied for about 30 seconds, before the cantilever was fully retracted. In order to ensure a firm contact between the cell and the cantilever, it was left to rest in this position for 10 minutes. Standard parameters were chosen for the SCFM measurements. Approach and retract velocities were fixed at 5 *μ*m/s using a set-point force of 1 nN.

**Figure 2:**
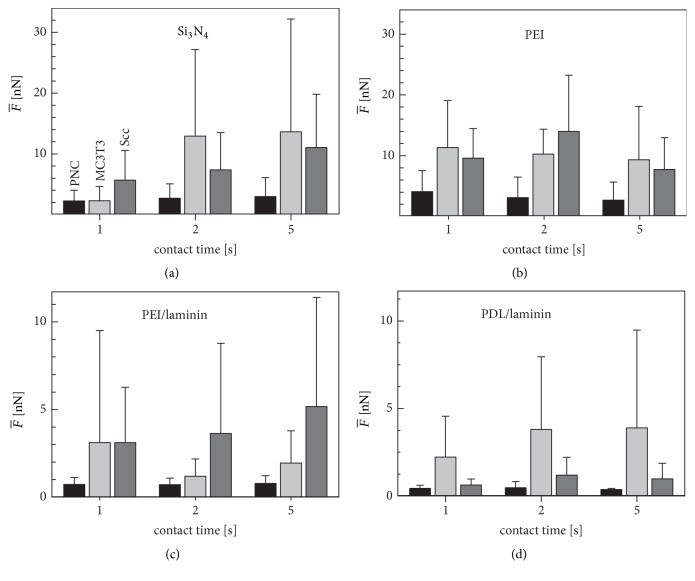
Mean measurement MDFs (${\overset{-}{F}}_{n,s,t}$) of the three cell types, PNCs (black, n=12), MC3T3 (light gray, n=14), and Scc (dark gray, n=10), on the silicon nitride reference surface for contact times of 1, 2, and 5 seconds (a). *F*_*n*,*s*,*t*_ on PEI, PEI/laminin, and PDL/laminin surfaces for the same cells (PNCs, n=10; MC3T3, n=9; Scc, n=9) are presented in (b), (c), and (d). The measurement data are given in the supplementary material (Table S1). The statistical differences in the characteristic MDF behavior of the cell types were analyzed by two-way variance analyses (Table 1).

The freshly attached cell was aligned above the pure silicon nitride surfaces to determine its individual reference MDF. Twenty force-distance curves were recorded with a randomly chosen contact time of 1, 2, or 5 seconds (Figure 2(a)), before a differently coated surface was randomly chosen and probed 20 times for the same contact time. To ensure that each curve was recorded at an untouched coating location, the cantilever was shifted 25 *μ*m for each new force-distance curve in a predefined 5x5 grid. A measurement cycle was completed after all the surfaces had been probed. The next measurement cycle was started with the reference surface and a different contact time. After all the contact times in the third cycle had been processed, the cantilever with the cell was checked microscopically before it was discarded. Images of the cantilevers with cells before and after measurements are given in the supplementary material.

However, it was not always possible to capture complete datasets for all surfaces with the same cell. If a cell showed strongly decreasing MDFs for a certain coating, the experiment was aborted. In these cases, only measurements with the previously measured coatings were considered in the data evaluation. The experiments were continued with a fresh cell attached to a new cantilever. This procedure resulted in a higher number of cells being measured on the reference surface.

### 2.6. Handling of Measurement Data

The following considerations were made for each of the three cell species studied. First, let *F*_*n*,*s*,*t*_ be the MDF of an individual cell, where the indices* n*,* s*, and* t* stand for number of the cell, the surface type, and the contact time. The MDF was obtained for the reference surface* s*=0 (silicon nitride) or one of the three surfaces* s*=1 (PEI), s=2 (PEI/laminin), or s=3 (PDL/laminin) for the contact times* t*=1, 2, or 5 seconds.

In order to improve the comparability of the MDFs on the different surfaces, the MDF of a single cell measured on the coated surfaces can be normalized to those measured on the reference surface (*s*=0). In such a case, different normalization options can be contemplated, each of them highlighting particular differences. In order to consider the influence of the contact time on the MDF for the three cell species, the MDF of each cell (*F*_*n*,*s*,*t*_) was divided by the corresponding reference (*F*_*n*,0,5_), which is the MDF on the reference surface for a contact time of 5 seconds. This yields a dimensionless relative parameter with reduced effects of individual cell properties, such as cell size:

(1) $$\begin{array}{c} \Phi_{n,s,t}^{5sec}=\frac{F_{n,s,t}}{F_{n,0,5}} \end{array}$$

Averaging provides the mean relative MDF for the cell type being studied:

(2) $$\begin{array}{c} {\overset{-}{\Phi }}_{s,t}^{5sec}=\frac{1}{n}\underset{n=1\text{..}n}{\sum }\Phi_{n,s,t}^{5sec} \end{array}$$

Nevertheless, this parameter equilibrates fundamental disparities in the MDF strengths of the different cell species. In order to restore this feature and to highlight the effects of the different coatings on the MDFs of the different cell types, Φ_*n*,*s*,*t*_^5*sec*^ was transformed into the specific MDF *F*_*n*,*s*,*t*_^spec^ of the cells of the considered species. This was achieved by multiplying the Φ_*n*,*s*,*t*_^5*sec*^ of each cell by ${\overset{-}{F}}_{0,5}$, the mean MDF on the reference surface for a five-second contact:

(3) $$\begin{array}{c} F_{n,s,t}^{spec}=\Phi_{n,s,t}^{5sec}{\overset{-}{F}}_{0,5}=\Phi_{n,s,t}^{5sec}\frac{1}{n}\underset{n=1\text{..}n}{\sum }F_{n,0,5} \end{array}$$

Averaging over all cells yields the specific mean MDF for each surface and contact time:

(4) $$\begin{array}{c} {\overset{-}{F}}_{s,t}^{spec}=\frac{1}{n}\underset{n=1\text{..}n}{\sum }F_{n,s,t}^{spec} \end{array}$$

Another normalization method emphasizes the influence of a particular coating on the interaction with a particular cell type. The relative parameter Φ_*n*,*s*,*t*_^*τ*^ of cell number n was obtained for s = 1, 2, and 3 and for t = 1, 2, and 5 s by dividing the MDFs of each contact time of a cell *F*_*n*,*s*,*t*_ by *F*_*n*,0,*t*_, the MDF on the reference surface for the same contact time t. Averaging over n cells gives us

(5) $$\begin{array}{c} {\overset{-}{\Phi }}_{s,t}^{\tau }=\frac{1}{n}\underset{n=1\ldots n}{\sum }\Phi_{n,s,t}^{\tau }=\frac{1}{n}\underset{n=1\ldots n}{\sum }\frac{F_{n,s,t}}{F_{n,0,t}} \end{array}$$

This dimensionless parameter has been identified by superscript *τ*. In order to obtain dimensional, contact-time-specific MDFs for a particular cell type, the relative MDF Φ_*n*,*s*,*t*_^*τ*^ was multiplied by the mean MDF on the reference surface for the considered contact time. Averaging over n cells yields

(6) F−s,tτspec=1n∑n=1..nΦn,s,tτF−0,t=1n∑n=1..nΦn,s,tτ1n∑n=1..nF−n,0,t

## 3. Results

### 3.1. Cell Attachment to the Cantilever

The same poly-dopamine coating was used to attach single cells of all types to the cantilever. During polymerization under alkaline conditions, poly-dopamine forms thin layers and adheres to almost any surface [42]. However, the mechanisms of polymerization and the mechanisms of binding of the poly-dopamine layers to the cell surfaces and the cantilever surface are still being investigated [42, 43].

The thickness of the poly-dopamine layer on the cantilever depended on the incubation time [42]. We found that reliable immobilization of our cells on the cantilever was hardly possible with incubation times of much shorter than 25 minutes. However, for incubation times longer than 25 minutes, the reflectivity of the cantilever decreased, as the diminishing total intensity of the reflected laser signal showed. We therefore considered the polymerization time of 25 min to be optimal. After calibrating the coated cantilevers, single PNCs, MC3T3, or Scc cells were attached to the front of the cantilevers.

### 3.2. Measurement MDFs

Figure 2(a) summarizes the MDF results for the pure, uncoated silicon nitride reference surface. Of the three cell types, PNCs showed the lowest MDFs on all surfaces except MC3T3 cells on silicon nitride for 1-second contact time (Figure 2(a)). As expected, the MDFs were generally increased for longer contact times, except for the MC3T3 cells, which nevertheless showed a steep MDF increase as early as from 1 to 2 seconds (Figure 2(a), light gray bars).

**Figure 3:**
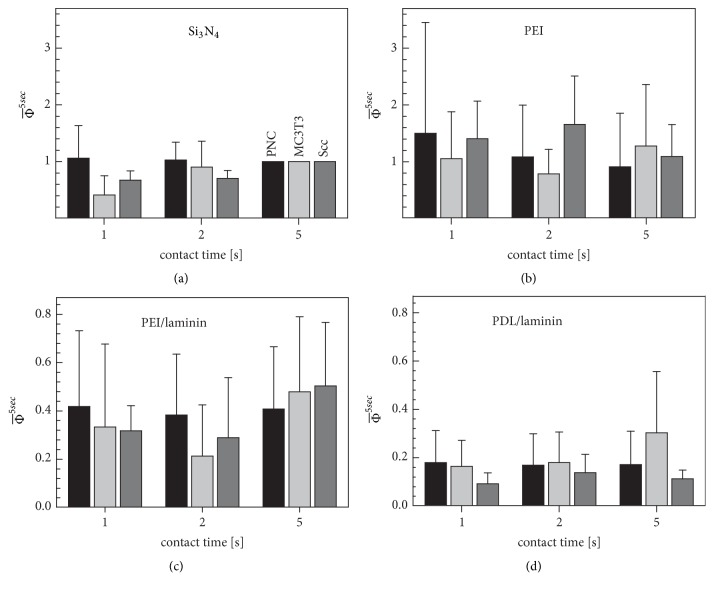
Mean relative MDFs (${\overset{-}{\Phi }}_{n,s,t}^{5sec}$) of PNCs (black), MC3T3 cells (light gray), and Scc cells (dark gray) on silicon nitride (a), PEI (b), PEI/laminin (c), and PDL/laminin (d) surfaces. Unit columns without scattering were obtained for the five-second MDFs on silicon nitride used as reference.

For one-second contact time, the MDFs on the PEI-coated surfaces were significantly increased compared to the silicon nitride surface for all cell types (cf. Figures 3(a) and 3(b)). Apart from the absolute MDFs, the temporal increase was faster for all other coatings than for silicon nitride. Please note that the MDFs of the Sccs on PEI increased between 1 and 2 seconds' contact time, but at 5 seconds the average MDF was less than the MDF at 2 seconds' contact time (Figure 2(b), dark gray bars).

The addition of laminin reduced the MDFs for all cell types compared to silicon nitride and PEI. To characterize the effect of the two base coatings on the laminin surface, we compared PEI/laminin with PDL/laminin. The measurement MDFs on the PEI/laminin surfaces were up to more than twice as high as that on PDL/laminin for PNCs and Scc cells, while this relationship was almost reversed for MC3T3 cells for contact times of 2 and 5 seconds.

The laminin density on the PDL and PEI coatings was quantified with fluorescence staining. The fluorescence detected on the PEI surface was approximately twice as high as on the PDL surface, which corresponds to twice the concentration of surface-bound laminin (see supplementary material).

### 3.3. Relative and Specific MDFs on Different Coatings

For further data interpretation, we normalized the data. The MDF of each cell for the differently coated surfaces (*F*_*n*,*s*,*t*_) was divided by *F*_*n*,0,5_, which is the MDF on the reference surface for 5 seconds' contact time (see (3)). The obtained relative MDF values were roughly comparable for all cell types on a given coating (Figure 3). On average, the standard deviation of the relative MDFs was reduced by 22.7% compared to the standard deviation of the measurement MDFs. Obviously, the relative MDFs on the coated surface were highest for PEI, lower for PEI/laminin, and lowest for PDL/laminin, which is largely consistent with the measurement MDFs (cf.  Figure 2 with Figure 3).

**Figure 4:**
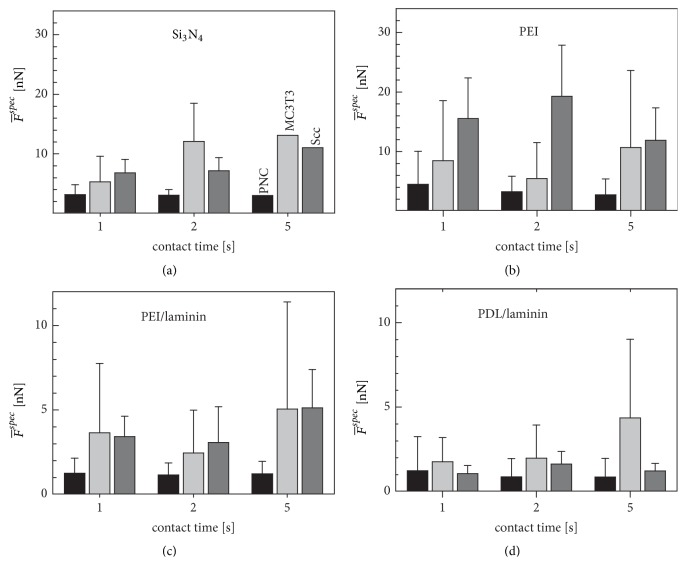
Mean specific MDFs (${\overset{-}{F}}_{s,t}^{spec}$) (see (4)) of PNCs (black), MC3T3 (light gray), and Scc (dark gray) on silicon nitride (a), PEI (b), PEI/laminin (c), and PDL/laminin (d) surfaces. The statistical differences in the characteristic MDF behavior of the cell types were analyzed by two-way variance analyses (Table 1).

To consider cell type-specific MDFs for the different coatings, we multiplied the relative MDF of each individual cell by the mean measurement MDF of its cell type for the five-second contact time on the reference surface (see (3)). The specific MDFs obtained (see (4)) generally maintain the reduced scattering of the relative MDFs (Figure 4).

Although there are clear differences between the measurement MDFs and the specific MDFs, the two parameters are clearly within the same force range, while the general MDF differences for the different coatings have been retained (cf.  Figure 2 with Figure 4).

A pronounced hierarchy between the cell types became visible on PEI at the contact times of 1 and 2 seconds. The specific MDFs on the PEI/laminin and PDL/laminin coatings were generally increased for PNCs, while the differences due to the different contact times changed only slightly within the measurement MDFs and within the specific MDFs. On the PEI/laminin coating, both MC3T3 and Scc cells each showed almost the same specific MDFs for each of the different contact times. The number of statistically significant differences (p≤ 0.05) increased from 11 (measurement MDFs) to 13 (specific MDFs) in the performed two-way variance analyses (Table 1). See the supplementary material for more detailed results.

While the introduction of the specific MDF did not improve the statistical significance of the PNC results, it was able to improve the significance for both MC3T3 and Scc cells. It should be emphasized that statistically significant differences between PEI/laminin and PDL/laminin existed only for the Scc cells and that no statistically significant differences were found for the specific MDFs of MC3T3 cells and PNCs (Table 1). Comparing the MDF-behavior of the three cell types, the PNCs showed a statistically significant different behavior from the MC3T3 and Scc cells, which behaved more similarly (see supplementary material).

## 4. Discussion

### 4.1. Relative and Specific MDFs

SCFM is a standard method for investigating cell-surface interactions on a single cell level [35, 44]. Even though SCFM is an elegant procedure, SCFM measurements have not yet become a standard in the search for surface coatings for biomedical applications [11]. In general, the standard deviation of SCFM results is high due to the biological variability of individual cells with respect to the characteristic being studied [13]. This problem makes it all the more important to use the same single cell to probe many different surfaces. Another problem is that SCFM only allows initial adhesion forces to be detected, since longer adhesion times with the resulting stronger adhesions of the measuring cell to the examined surfaces may lead to the cell being detached from the cantilever.

Pure silicon nitride surfaces served as reference in our experiments. Since these surfaces are smooth, homogeneous, and reproducible, they introduced very few additional parameters for normalization. Relative MDFs helped to alleviate the problem of scattering of individual cell properties. Here, the MDF of each cell for the differently coated surfaces was divided by the MDF of the 5-second contact time on the reference surface. As another way of normalizing the measurement MDFs, the MDF of each cell was normalized for each contact time on a given coating to the same contact time on the reference surface (see (5); see the supplementary material for the results). While this normalization method can provide information on the MDF differences caused by the different coatings, the method preferred here also retains the contact time dependencies. It assumes that the five-second MDF on the reference surface has approached a “plateau MDF.” We found that both methods usually reduced the standard deviations and increased the statistical significance, probably because the effects of certain cell properties such as cell size, contact area, or specific surface charge were eliminated.

When relative values only were calculated, the fundamental MDF differences between the cell types disappeared (Figure 3). The data shows that the three coatings induce different relative MDFs. Interestingly, these differences are very similar for each cell type, suggesting the same qualitative interaction and adhesion mechanisms for all cell types.

**Figure 5:**
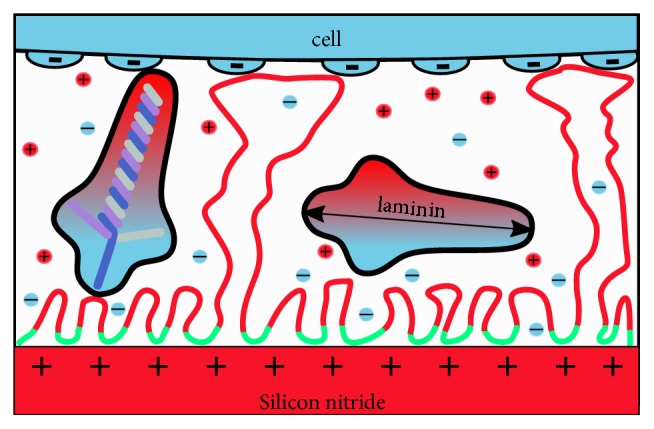
Sketch of a negatively charged cell surface in contact with a PEI/laminin-coated silicon nitride surface. Protonated amino groups generate the positive charge of the flexuous PEI polymer chain (red segments). The positive charges on the silicon nitride surface increase the pH over the double layer length and thus deprotonate the PEI polymer (green segments). The segments discharged are bound to the surface by van der Waals forces. Flexuous PEI loops with an approx. length of 100 nm can span the laminin layer and create adhesion between the surfaces. The maximum length of the elongated laminin protein is approx. 80 nm (double arrow).

The basic differences between the measurement MDFs were eliminated by applying (3) to the relative MDFs. The averaged specific MDFs obtained (see (4)) again show the cell-type specificity of the measurement MDFs (Figure 5). In contrast to the measurement MDFs, specific MDFs have the advantage that specific properties of the individual cells that influence the measurements, such as cell size, contact area, density of the contact-mediating charges, or molecular groups, are compensated in full or reduced in influence. Other groups minimized the influence of such properties, after optical measurement of cell size and contact area [11]. An additional advantage of our method could be that the influence of impurities in the measuring medium is compensated for, since we used the same cell in the same external medium for all surfaces.

Our normalization ensures that some unwanted effects that affect all cells in the same manner disappear in the relative MDFs, whereas those effects that depend on the surface coatings are visible in both the relative MDFs and the specific MDFs. We believe that specific MDFs are superior to the measurement MDFs when the focus is on the effects of coatings on cell populations. Moreover, as mentioned in the results section, the number of cells measured on the reference surface was higher than on the other surfaces. This directly increased the reliability of the reference MDFs and thus the reliability of the specific MDFs obtained. However, it may be worth comparing the MDF differences between cells of one type on the different coatings before and after normalization, as specific MDFs may mask certain cell-specific characteristics, such as possible differences between neurons and glia cells within the PNC population. In order to determine such differences by separating subpopulations, an analysis of the measurement MDFs on significantly more single cells would be necessary.

### 4.2. Surface Properties of Silicon Nitride and Coatings of PEI and PDL

Silicon nitride surfaces carry different chemical groups, most of which are positively charged at a pH of 7.4 [45]. The PEI polymer backbone consists of recurring units of aliphatic spacers and amine groups. The branches consist of the same monomeric units with primary, secondary, and tertiary amino groups, which are predominantly protonated and thus positively charged at pH 7.4.

In the literature, the chemical interaction of PEI with silicon dioxide (silica) has been investigated in more detail than in the case of silicon nitride [46]. The positively charged PEI molecules bind to silicon dioxide with a high affinity, though mainly through nonelectrostatic interactions. We find it very plausible that similar nonelectrostatic interactions may play a predominant role in the adsorption of PEI to the positively charged silicon nitride, since silicon dioxide and silicon nitride share similar crystal lattice structures on their surfaces (Figure 5). When high ionic strength is used, PEI coatings with long molecular loops are formed in a wide pH range, from pH 6 to 9. The loops protrude into the bulk water from a denser, surface-bound polymer layer [47]. After their formation, these structures are stable against pH fluctuations.

The type of PEI used here is more highly branched than the PDL polypeptide, resulting in a higher number of amino groups per M_w_. In their protonated form, these groups are the positive charge carriers for both polymers, indicating that the cells with the PEI coatings experience a higher spatial charge density and a stronger electrostatic interaction than those with the PDL coatings.

Like PEI, the PDL polypeptide is known to adsorb on negative surfaces by electrostatic interactions with strong nonelectrostatic contributions. For PDL, the surface layers may be packed in different densities [48]. We believe that the nonelectrostatic interactions provide the adsorption mechanism of PDL to the positively charged silicon nitride surfaces similar to the adsorption of PEI. For PDL coatings, we could not find any information on possible loop formations similar to the PEI loops.

We presume that the positive charge densities of the silicon nitride and PEI surfaces are comparable, resulting in the comparable MDFs at contact times of 2 and 5 seconds (Figure 3). This results in relative MDFs scattering around unity for silicon nitride for these contact times (Figure 3(a)). Interestingly, for MC3T3, lower MDFs are registered on silicon nitride for the one-second contact time (cf.  Figure 4(a)with 4(b)). We believe that the cellular surface molecules can form bonds faster with the PEI loops than with the flat silicon nitride surface [47]. This effect seemed to be particularly pronounced in MC3T3 cells.

### 4.3. Laminin Effect on PEI and PDL Coated Surfaces

Here, we found that silicon nitride and PEI induced similar MDFs, which were significantly higher than on the laminin-finished coatings (Figures 4 and 5). In general, MDFs were higher on PEI/laminin than on PDL/laminin surfaces. Since specific binding mechanisms can be excluded for both surfaces, we assume that the MDFs detected are based on the accessible length of the PEI or PDL polymers and the higher spatial density of the charged groups in the PEI polymer.

Laminin-111 is a 900 kDa protein that adsorbs in flexible conformations. It can be assumed that the length of the PEI loops reaches the height of the adsorbed protein or is slightly longer (Figure 5). For all three cell types, the additional laminin coating reduces the MDFs compared to the pure silicon nitride surface or PEI coating, suggesting that laminin reduces the initial unspecific electrostatic interaction mechanisms. This also applies to PNCs, which are known to carry specific laminin-binding molecules [26, 27]. The specific protein-mediated binding mechanisms are only effective for times of much longer than one minute, suggesting that laminin acts as a geometric spacer in our experiments that prevents direct cell contact with charged surfaces.

Our fluorescence measurements showed that the laminin coverage on the PEI-coated surfaces was twice as high as on the PDL-coated surfaces. Accordingly, the still higher MDFs on PEI/laminin must be produced by either a significantly higher number of protonable amino groups of PEI or their higher accessibility. The prerequisite for the higher accessibility would be that the loops described above project into the bulk volume between the laminin proteins (Figure 5). A possible second-order effect would be that the charge of the underlying coating polarizes the laminin molecule and induces positive charges on the opposite side of the protein, which then interact with the cellular surface charges. This second-order effect would probably be stronger on PEI than on PDL.

### 4.4. Cell Type Specific Properties

For eukaryotic cells, most adhesion models describe the initial phase as an electrostatically driven process in which electrostatic interactions, dipole-dipole interactions, and van der Waals forces bind the cells to a surface [34, 35]. These mechanisms also apply to prokaryotic cells [36]. For eukaryotic cells, specific integrin interactions play a role only after more than 10 minutes [34, 35].

Our relative MDFs show that four different surfaces induced approximately the same relative forces for each cell type although the MDF magnitudes were strongly dependent on the cell type (cf. Figures 4 and 5). This strongly suggests that the adhesion mechanisms were the same for all cell types, which makes the electrostatic explanation very likely.

In the SCFM-retract phase, the contact points of eukaryotic cells are loaded with constant force over the time of tether extraction, which leads to a certain tear-off probability [44, 49]. For bacteria, the cell wall prevents them from forming membrane tethers, which are elongated with a constant load force. The resulting shorter load times for the molecular contact points result in rapidly increasing forces in the force-distance curves, an increasing probability of tearing off and higher MDF values.

We are not aware of any reports on specific interactions of laminin with MC3T3 or Scc cells. Interestingly, these cells show very similar specific MDFs on PEI/laminin. On PDL/laminin surfaces, their specific MDFs are differently reduced, suggesting that the PDL base coating discriminates against Scc cells.

For eukaryotic cells, the strong surface adhesion is a prerequisite for their proliferation on the respective surface [50]. For example, neuronal networks on PDL/laminin-coated surfaces show clustered cell growth, while they form relatively homogeneous monolayers on pure PEI surfaces ([51] and our own unpublished data). A more homogenous cell distribution and higher neuronal activity were also observed on PEI/laminin-coated multi-electrode arrays, which is consistent with the slightly higher forces induced by PEI/laminin compared to PDL/laminin [38].

## 5. Conclusions

Our results are in line with cell adhesion models in which the initial phase of adhesion is dominated by the electrostatic interaction between negatively charged cell membranes and positively charged surfaces, without specific adhesion molecules playing a role. For prokaryotic cells, it is known that electrostatic surface interactions are crucial for the successful formation of colonies [36]. In previous experiments with MC3T3 cells, we found that their MDFs were elevated at alkaline pH, which correlated with increased proliferation in cell culture experiments [33]. Nevertheless, we are not sure about the general strength of the predictive power of initial adhesion for the fate of proliferation of prokaryotic and eukaryotic cells, since many different mechanisms only become effective after the initial phase. To shed light on this problem, we are currently preparing a manuscript in which we compare SCFM results with FluidFM results. With this method, the much stronger detachment forces of individual cells after days of growth on a surface can be measured [52].

Silicon nitride is a common material for* in vitro* cell monitoring devices, but it is not yet as widely used in standard biological applications [3, 53, 54] although it does have similar MDF-enhancing properties to the common cell culture coating PEI.

Laminin reduced the MDFs for all cell types by covering parts of the underlying positively charged surfaces. Interestingly, a similar reduction in initial MDFs was observed in L929 cells after fibronectin coating of silicon oxide layers (data not published). Although the coverage for PEI was higher than for PDL, the MDFs for PEI/laminin were still higher than for PDL/laminin, which can be explained by the long loops that PEI may make and which can bridge the laminin layer. Interestingly, MC3T3 and Scc cells show very similar specific MDFs on PEI/laminin. Their specific MDFs are reduced by different degrees on PDL/laminin surfaces, suggesting that the PDL base coating discriminates against Scc cells. In this context, it could be speculated that the electrostatic charge interaction with the PEI loops is more important for the initial adhesion of the smaller prokaryotic cells than for eukaryotic cells.

In order to highlight the specificities of the cell types and to increase the statistical significance, we normalized the MDFs to a reference surface. The transformation of the relative MDFs obtained into specific MDFs led to a new parameter that can also be easily applied to other SCFM data.

## Figures and Tables

**Table 1:** Result of the two-way ANOVA analysis of measurement MDFs (${\overset{-}{F}}_{n,s,t}$) and specific MDFs (${\overset{-}{F}}_{s,t}^{spec}$) for each cell type separately taking the three contact times into consideration. Significance levels are marked for p≥ 0.05 (none), p≤ 0.05 (*∗*), and p≤ 0.01 (*∗∗*) and separated by a slash (${\overset{-}{F}}_{n,s,t}/\;{\overset{-}{F}}_{s,t}^{spec}$).

Statistical differences	PNCs	MC3T3	Scc
silicon nitride vs. PEI	-/-	-/-	-/*∗∗*
silicon nitride vs. PEI/laminin	*∗∗*/*∗∗*	*∗*/*∗*	*∗*/*∗∗*
silicon nitride vs. PDL/laminin	*∗∗*/*∗∗*	*∗*/*∗∗*	*∗∗*/*∗∗*
PEI vs. PEI/laminin	*∗∗*/*∗∗*	*∗*/*∗∗*	*∗∗*/*∗∗*
PEI vs. PDL/laminin	*∗∗*/*∗∗*	*∗*/*∗∗*	*∗∗*/*∗∗*
PEI/laminin vs. PDL/laminin	-/-	-/-	-/*∗∗*

## Data Availability

The experimental MDFs for each cell separately are summarized in an excel file (Table S1) in the supplementary material.

## Supplementary Materials

Supplementary Materials Tables S2a-e and S3a-e summarize the results of two-way ANOVA analyses of the statistical differences in the measurement MDFs and in the specific MDFs. Figures S1 and S2 show the results of the alternative normalization method according to equations (5) and (6).
