# Supplementary material for: Surface Coatings Modulate the Differences in the Adhesion Forces of Eukaryotic and Prokaryotic Cells as Detected by Single Cell Force Microscopy
Source: Int J Biomater. 2019 Apr 1;2019:7024259. doi: 10.1155/2019/7024259 (PMC6463582; doi:10.1155/2019/7024259)
Supplement: Supplementary Materials — Tables S2a-e and S3a-e summarize the results of two-way ANOVA analyses of the statistical differences in the measurement MDFs and in the specific MDFs. Figures S1 and S2 show the results of the alternative normalization method according to equations (5) and (6). [file 7024259.f1.zip › 7024259.f1/7024259.f2.pdf]

Supplementary material

The significance levels are marked for p≥ 0.05 (none), p≤ 0.05 (*) and p≤ 0.01 (**) in the following.

Measurement data

The Excel table (Table S1) summarizes the mean maximum detachment forces (MDFs) of at least 20 experimentally determined force-displacement curves for each measured cell.

Table S1: MDF measurement data. The single cells are numbered from 1 to n with Scc cells (n=10), MC3T3 cells (n=14) and PNCs (n=12). The units for the MDF values and contact times are nN and seconds, respectively.

Results of the alternative normalization method (Eq. 5)

Figure S1 presents the mean relative MDFs ($\overline{\Phi}^{\tau}_{n,s,t}$) resulting from the alternative normalization method according to Eq.5, which was mentioned in the main text. In this method, the MDF of each individual cell is divided by the MDF for the same contact time on the reference surface. It should be noted that the contact time dependence of the measuring MDFs for the differently coated surfaces is missed in these results, since each contact time was individually normalized to silicon nitride reference.

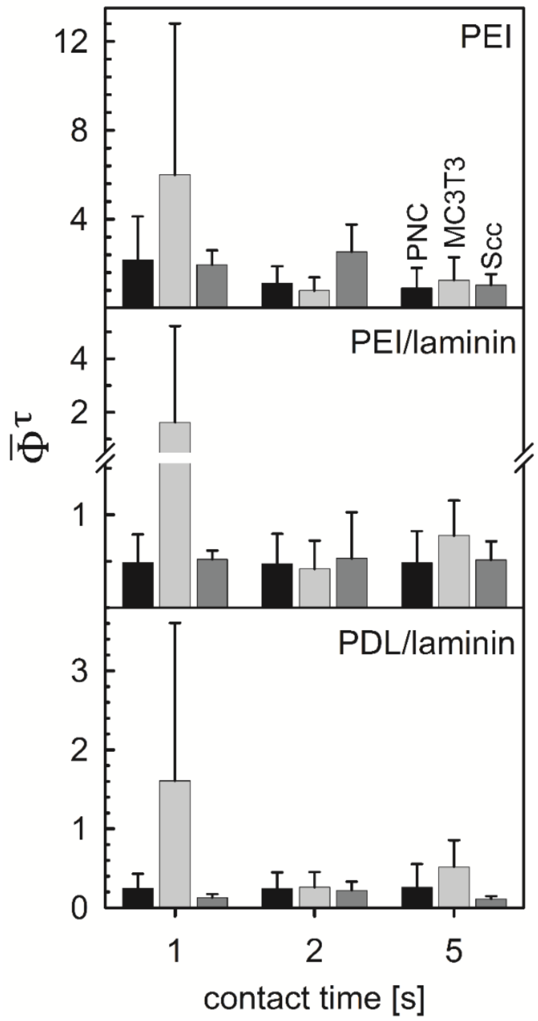

Figure S1: Mean relative MDFs ($\overline{\Phi}^{\tau}_{n,s,t}$) for the alternative normalization method of Eq.5. The PNCs are black, MC3T3 light gray, and Scc dark gray bars.

Figure. S2 presents the Specific MDFs ($\bar{F}_{s,t}^{\tau spec}$) resulting from the alternative normalization method. For the specific MDFs, the relative MDF of each individual cell was multiplied with the mean measurement MDF on the reference surface for the respective contact time (see eq. 6).

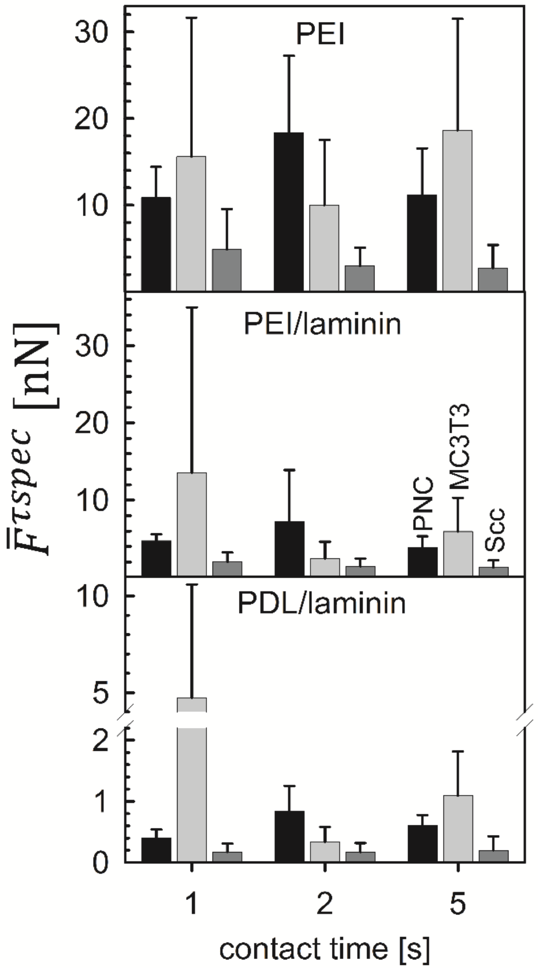

Figure S2: *Specific MDFs ($\bar{F}_{s,t}^{\tau spec}$) of PNCs (black), MC3T3 (light gray), and Scc (dark gray) o nPEI, PEI/laminin, and PDL/laminin surfaces.*

Results of the two-way ANOVA of the measurement MDFs

Table S2a: Two-way ANOVA analysis of the measurement MDFs to compare the different cell types on the surface coatings.

Statistical differences	silicon nitride	PEI	PEI/laminin	PDL/laminin
MC3T3 vs. PNCs	**	**	-	-
MC3T3 vs. Scc	-	-	-	-
Scc vs. PNC	**	**	-	-

Table S2b: Two-way ANOVA analysis of the measurement MDFs of primary neuronal cells (PNC).

Statistical differences (PNC)	1 s contact time	2 s contact time	5 s contact time
silicon nitride vs. PEI	-	-	-
silicon nitride vs. PEI/laminin	-	-	-
silicon nitride vs. PDL/laminin	-	-	-
PEI vs. PEI/laminin	*	-	*
PEI vs. PDL/laminin	*	-	*
PEI/laminin vs. PDL/laminin	-	-	-

Table S2c: Two-way ANOVA analysis of the measurement MDFs of osteoblast like cells (MC3T3).

Statistical differences (MC3T3)	1 s contact time	2 s contact time	5 s contact time
silicon nitride vs. PEI	-	-	
silicon nitride vs. PEI/laminin	-	*	*
silicon nitride vs. PDL/laminin	-	*	*
PEI vs. PEI/laminin	-	-	-
PEI vs. PDL/laminin	-	-	-
PEI/laminin vs. PDL/laminin	-	-	-

Table S2d: Two-way ANOVA analysis of the measurement MDFs of staphylococcus capitis sub. capitis (Scc).

Statistical differences (Scc)	1 s contact time	2 s contact time	5 s contact time
**silicon nitride vs. PEI**	-	-	-
**silicon nitride vs. PEI/laminin**	-	-	-
**silicon nitride vs. PDL/laminin**	-	-	-
**PEI vs. PEI/laminin**	-	-	-
**PEI vs. PDL/laminin**	*	-	-
**PEI/laminin vs. PDL/laminin**	-	-	-

Results of the two-way ANOVA of the specific MDFs

Table S3a: Two-way ANOVA analysis of the specific MDFs ($\bar{F}_{s,t}^{spec}$) to compare the different cell types on the surface coatings.

Statistical differences	silicon nitride	PEI	PEI/laminin	PDL/laminin
MC3T3 vs. PNCs	**	**	*	*
MC3T3 vs. Scc	**	-	-	*
Scc vs. PNC	**	**	*	-

Table S3b: Two-way ANOVA analysis of the specific MDFs ($\bar{F}_{s,t}^{spec}$) of primary neuronal cells (PNC) for each contact time separately.

Statistical differences (PNC)	1 s contact time	2 s contact time	5 s contact time
silicon nitride vs. PEI	-	-	-
silicon nitride vs. PEI/laminin	-	-	-
silicon nitride vs. PDL/laminin	-	-	-
PEI vs. PEI/laminin	*	-	-
PEI vs. PDL/laminin	*	-	-
PEI/laminin vs. PDL/laminin	-	-	-

Table S3c: Two-way ANOVA analysis of the specific MDFs ($\bar{F}_{s,t}^{spec}$) of osteoblast like cells (MC3T3) for each contact time separately.

Statistical differences (MC3T3)	1 s contact time	2 s contact time	5 s contact time
silicon nitride vs. PEI	*	-	
silicon nitride vs. PEI/laminin	-	*	-
silicon nitride vs. PDL/laminin	-	*	*
PEI vs. PEI/laminin	*	*	*
PEI vs. PDL/laminin	*	*	*
PEI/laminin vs. PDL/laminin	-	-	-

Table S3d: Two-way ANOVA analysis of the specific MDFs ($\bar{F}_{s,t}^{spec}$) of staphylococcus capitis sub. capitis (Scc) for each contact time separately.

Statistical differences (Scc)	1 s contact time	2 s contact time	5 s contact time
silicon nitride vs. PEI	*	*	-
silicon nitride vs. PEI/laminin	-	*	*
silicon nitride vs. PDL/laminin	*	*	*
PEI vs. PEI/laminin	*	*	*
PEI vs. PDL/laminin	*	*	*
PEI/laminin vs. PDL/laminin	-	-	-

Two-way ANOVA analysis for differences in the behavior of the cell types

Table S4: Two-way ANOVA analysis for the cell types.

Statistical differences	measured	specific
PNC vs. MC3T3	**	**
PNC vs. Scc	**	**
Scc vs. MC3T3	-	-

Laminin surface coverage

Fluorescence microscopy was used to compare the laminin densities on PEI and PDL base coatings. The surface-bound laminin was bound by a primary rabbit antibody and detected by a secondary antibody labelled with a fluorophore (see Materials and Methods).

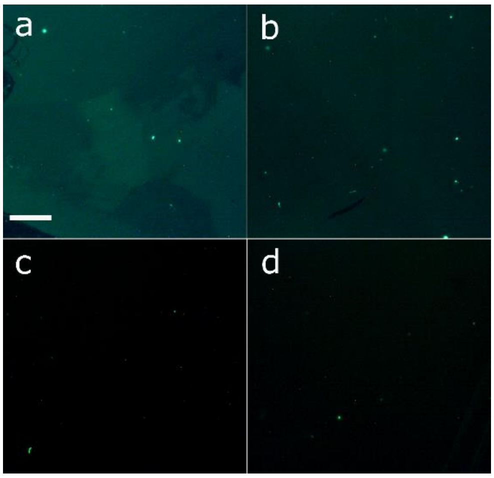

Figure S3: Fluorescence images of the PEI (a) and PDL (b) surfaces, which were immuno-stained with fluorescence-labelled laminin antibodies. In order to compensate for the auto-fluorescence of the coatings without laminin, PEI (c) and PDL (d) surfaces were also inspected. White bar: 50 µm.

Table S5: The amplitude of the green channel was averaged over approx. 0.64 mm^2 using the imageJ image analysis software (https://imagej.nih.gov/ij/). Four images were analyzed per coating.

	amplitude of green channel	standard deviation
PEI (background)	3.4	±0.03
PDL (background)	2.1	±0.08
PEI-laminin	22.2	±2.71
PDL-laminin	11.4	±1.10

Cells attached to a cantilever before and after a complete cycle of measurements

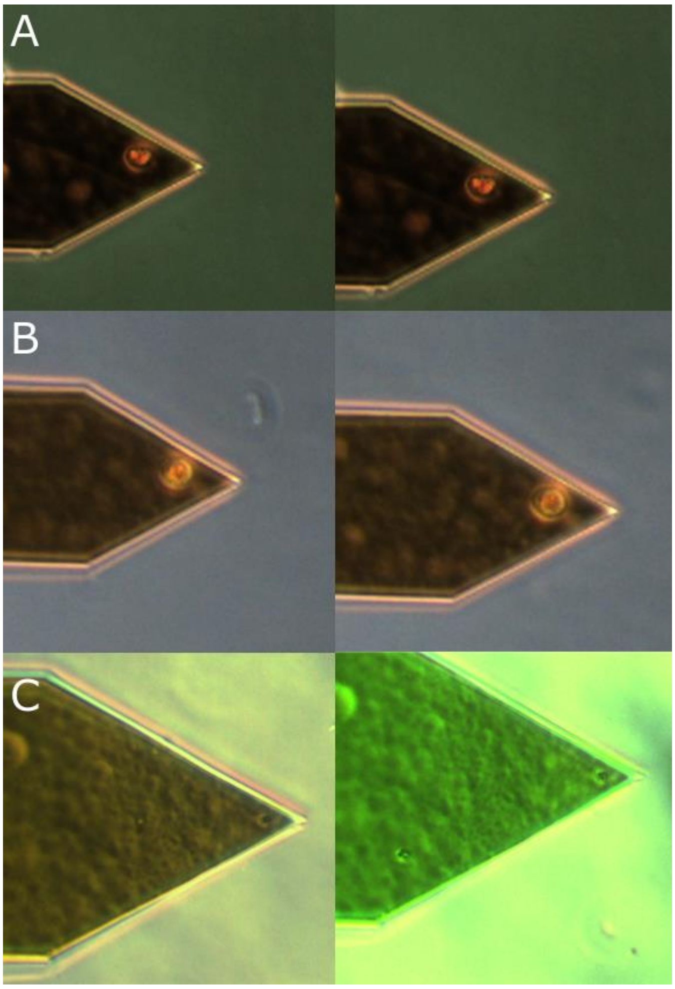

Figure S3: Representative microscopic of the three cell types PNCs (A), MC3T3 (B) and Scc cells attached to the cantilever before(left) and after (right) a complete cycle of measurements.
